# Projected costs associated with school-based screening to inform deployment of Dengvaxia: Vietnam as a case study

**DOI:** 10.1093/trstmh/try057

**Published:** 2018-07-03

**Authors:** Hugo C Turner, Bridget A Wills, Motiur Rahman, Hoang Quoc Cuong, Guy E Thwaites, Maciej F Boni, Hannah E Clapham

**Affiliations:** 1 Oxford University Clinical Research Unit, Wellcome Trust Major Overseas Programme, Ho Chi Minh City, Vietnam; 2 Centre for Tropical Medicine and Global Health, Nuffield Department of Medicine, University of Oxford, Oxford, UK; 3 Pasteur Institute, Ho Chi Minh City, Vietnam; 4 Center for Infectious Disease Dynamics, Department of Biology, Pennsylvania State University, University Park, Pennsylvania, USA

**Keywords:** cost, dengue, Dengvaxia, screening, serosurvey, vaccine

## Abstract

**Background:**

After new analysis, Sanofi Pasteur now recommends their dengue vaccine (Dengvaxia) should only be given to individuals previously infected with dengue and the World Health Organization’s recommendations regarding its use are currently being revised. As a result, the potential costs of performing large-scale individual dengue screening and/or dengue serosurveys have become an important consideration for decision making by policymakers in dengue-endemic areas.

**Methods:**

We used an ingredients-based approach to estimate the financial costs for conducting both a school-based dengue serosurvey and school-based individual dengue screening within a typical province in Vietnam, using an existing commercial indirect immunoglobulin G enzyme-linked immunosorbent assay kit. This costing is hypothetical and based on estimates regarding the resources that would be required to perform such activities.

**Results:**

We estimated that performing a school-based individual screening of 9-year-olds would cost US$9.25 per child tested or US$197,827 in total for a typical province. We also estimated that a school-based serosurvey would cost US$10,074, assuming one class from each of the grades that include 8- to 11-year-olds are sampled at each of the 12 selected schools across the province.

**Conclusions:**

The study indicates that using this vaccine safely on a large-scale will incur noteworthy operational costs. It is crucial that these be considered in future cost-effectiveness analyses informing how and where the vaccine is deployed.

## Introduction

Dengue is a mosquito-borne viral disease endemic in at least 100 countries across Asia, the Pacific, the Americas, Africa and the Caribbean.^1^ A recent study estimated that approximately 390 million dengue infections occur per year, of which 96 million manifest clinically.^2^ The spectrum of clinical presentations of symptomatic cases is broad, ranging from a brief febrile illness to severe and potentially fatal disease.^3^ Many symptomatic dengue cases do not get clinically diagnosed and the number of symptomatic cases is typically underreported.^4^ Dengue has four serotypes, and a second infection with a heterologous serotype to the first is more likely to be severe than the first infection.^3,5^ There is also notable heterogeneity in the level of dengue transmission between and within countries, depending on environmental factors.^2,6^

The first dengue vaccine (Dengvaxia, Sanofi Pasteur, Lyon, France) was licensed in December 2015.^7^ The World Health Organization (WHO) initial recommendations for how the vaccine should be deployed were based on advice from the WHO’s Strategic Advisory Group of Experts (SAGE) on immunization, published in July 2016.^8^ To date, the vaccine has been approved by regulatory authorities in 19 countries and it has been introduced in public immunization programs in The Philippines and Brazil.^7,9^ In the WHO’s position paper, the initial recommendation was that Dengvaxia should only be used in those ≥9 y of age, and only in ‘high-burden’ areas, defined in this context as areas where the seroprevalence in the group targeted for vaccination ideally exceeds 70% and is not below 50%.^8^ These recommendations were due in part to observations from the Dengvaxia phase 3 trials, indicating that the vaccine was associated with an increased risk of developing severe/hospitalized dengue in recipients younger than 9 y of age.^7,10–15^ Based on the trial data available at the time, there was uncertainty whether this increased risk was due to the recipient’s age or their serostatus when vaccinated, that is, whether or not they had experienced a dengue infection before receiving the vaccine.^7^ The SAGE considered further research into the safety of Dengvaxia in seronegative individuals a high priority and the WHO requested that Sanofi Pasteur provide more data on this issue.^7,8,16,17^

Sanofi Pasteur subsequently performed additional laboratory testing and in November 2017 confirmed that vaccine recipients who were inferred to have had no prior dengue infection (i.e., those seronegative at baseline) had a significantly higher risk of hospitalization/severe dengue compared with the unvaccinated trial participants, regardless of their age.^18^ Possible biologic explanations for this have been outlined previously.^19^ Sanofi Pasteur proposed that national regulatory agencies update the prescribing information to request that health care professionals assess the likelihood of prior dengue infection in an individual before vaccinating.^18^

In response to this new analysis, in April 2018 the SAGE updated their recommendations, stating that the preferred option for countries considering using Dengvaxia is to use a pre-vaccination screening strategy where only dengue seropositive individuals are vaccinated.^20^

It is therefore important that the potential costs of performing a large-scale individual dengue screening be evaluated. Given that dengue transmission varies significantly between different areas within countries, and that it is not currently possible to accurately infer the transmission intensity of an area using only the number of reported cases, deciding if and where screening should be undertaken will not be straightforward. One option might be to use dengue serosurveys to identify high-risk areas within a particular country or region, which could then be subsequently targeted with an individual screening–based vaccination strategy.

In this study we investigate the potential resources and costs required to perform two different dengue screening strategies, using Vietnam as a case study. It is important to highlight that this costing is hypothetical and based on estimates regarding the resources that would be required to perform such activities in the context of Vietnam.

## Materials and methods

### Study location and methodology

**Figure 1.**
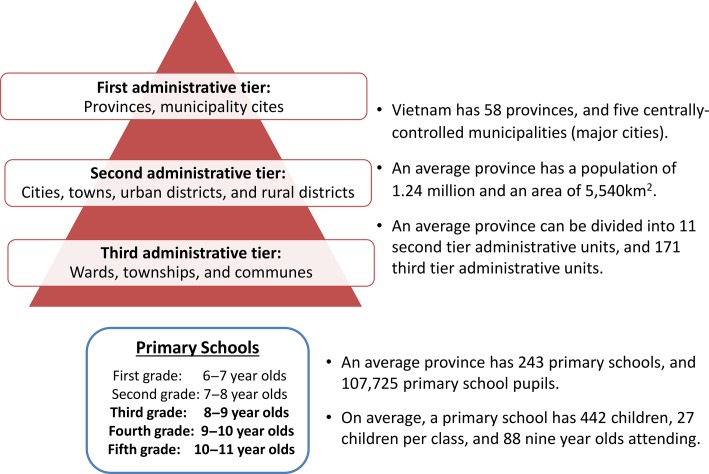
Overview of the administrative structure and educational system in Vietnam. The quoted figures pertain only to provinces and not the municipality cities. Based on data from the General Statistics Office of Vietnam.^21^

**Figure 2.**
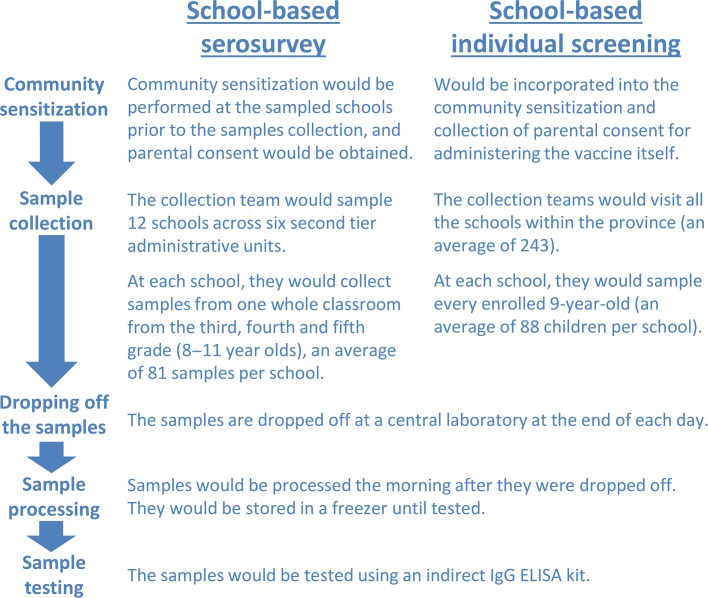
Schematic summary of the implementation of the serosurvey and individual screening.

For the purposes of this case study, we estimated the financial costs for conducting a school-based dengue serosurvey, in which only a sample of students are screened to obtain a population-level seroprevalence estimate, and school-based individual screening, where all the students within the targeted age group are screened within a typical province in Vietnam. An overview of the assumed characteristics of this hypothetical province is presented in Figure 1 and is based on averaged data from the general statistics office of Vietnam;^21^ a summary of the strategies and assumptions is shown in Figure 2 and Table 1.

**Table 1.** Summary of the key assumptions regarding the implementation of the serosurvey and individual screening

Variable	School-based serosurvey	School-based individual screening
Number of schools sampled within the province^a^	12	243
Number of children sampled per school^a^	81: one class from the third, fourth and fifth grades	88: the average number of 9-year-olds per school
Total number of children sampled	972	21 384
Number of schools sampled per day	1	1
Number of sample collection teams	1	2
Number of vehicles per team	2	2

^a^Based on data from the General Statistics Office of Vietnam.^21^ We assumed that private schools would not be included in the sampling.

#### School-based dengue serosurvey design

We based this on the recently published WHO dengue serosurvey guide.^22^ Due to the spatial variation in dengue transmission across Vietnam,^23^ we assumed that the second level of the three administrative tiers would be the main unit considered for the serosurvey (the level below provinces and municipality cities) (Figure 1). The serosurvey guide recommends that the administrative units considered should be subdivided into three dengue burden strata (highest, middle and lowest burden), based on their predicted seroprevalence (described further in the WHO’s dengue serosurvey guide^22^). However, the guide does not specifically state which strata should be sampled, since the selection depends on the relevant country’s desired vaccination strategy.^22^ For this study, we assumed that only the administrative units predicted to be within the highest dengue burden strata would be sampled (referred to as a highly regional targeted survey strategy^22^).

We assumed that at each school chosen, one classroom from the third, fourth and fifth grade would be sampled, consisting of children 8–9, 9–10 and 10–11 y of age, respectively. Although the serosurvey guide also recommends that 12-year-olds should be sampled,^22^ we assumed that this would not be feasible in Vietnam, as primary school ends at age 11 y and therefore 12-year-olds would be in a different school. We also assumed that private schools would not be included in the sample. Using the WHO’s dengue serosurvey sample size calculator,^24^ we estimated that six of the second-level administrative units (i.e. cities, towns, districts; Figure 1) would need to be sampled within the province, with two schools being sampled per unit.^22^ This corresponded to 12 schools and 972 children being sampled in total (81 per school) (Table 1). For this calculation, we took the estimated average primary school class size in Vietnam as 27^21^ (Figure 1 and Table 1). The parameters for the sample size calculator^24^ were informed by the serosurvey guide:^22^ the desired precision (i.e., the width of the confidence interval around the estimated mean seroprevalence) was set to be ±10%, the assumed mean seroprevalence was set to 70% and the intracluster/class correlation coefficient was set to 0.1. Based on these parameters, the design effect (DEFF) of the serosurvey was 3.5 (Box 1).^24^ We varied the key parameters of the sample size calculation within the sensitivity analysis (Table 2).

**Box 1.** Glossary

**Annualization:** The process of spreading the value of a capital resource over its useful lifespan resulting in an annualized cost. The simplest annualization method is straight-line depreciation, where the current cost of the capital resource is divided by its useful lifespan.
**Capital resources:** Resources that have useful lives greater than 1 y (such as laboratory equipment).
**Design effect (DEFF):** An inflation factor that reflects how much larger the survey needs to be because it does not have a simple random sample.^24^ For example, the clustering of students within the same schools makes the survey statistically less efficient.^24^ If the survey’s DEFF is equal to three, three times as many students are required to achieve the same precision as a simple random sample.^24^
**Economic costs:** Represent the full value of all resources used, including the value/opportunity cost of donated items. These are important when considering issues related to the sustainability and replicability of interventions.
**Economies of scale:** The reduction in the average cost per unit resulting from increased production/output. In this case it is the reduction in the cost per test as a result of increasing the number sampled.
**Financial costs:** Represent the accounting costs (i.e., actual amount paid) for a good or service.
**Fixed costs:** Costs that are not dependent on the quantity of output. In this case, costs that are incurred and do not change regardless of the total number sampled.
**Opportunity costs:** The value of a resource in its next best alternative use.
**Perspective:** The viewpoint from which the intervention’s costs and consequences are evaluated.
**Stepped-fixed costs:** Costs that are fixed for a particular level of activity/production but increase in a stepwise manner after exceeding a specific volume of activity/production. For example, these costs can be fixed per school sampled but variable in terms of the number of schools sampled.
**Variable costs:** Costs that vary in proportion to the quantity of output (i.e., the number of samples).

**Table 2.** A summary of the sensitivity analyses

Variable	Baseline assumption	Range explored
The number of schools sampled within the serosurvey	12	4–28
The level of additional community sensitization performed within the serosurvey	1 workday per school sampled	0–2 workdays per school sampled
Sample size per school	Serosurvey, 81; individual screening, 88	+10%
Whether 8-year-olds are included within the sample of the serosurvey	Yes (13 per school)	No
Intracluster/class correlation coefficient used within the sample size calculation for the serosurvey	0.1 (12 schools sampled)	0.15 (16 schools sampled)
Desired precision of the serosurvey (the width of the confidence interval around the estimated mean seroprevalence)	±10% (12 schools sampled)	±5 (44 schools sampled)
Proportion of the annualized laboratory equipment cost applied to our cost estimates.	Serosurvey, 5%; individual screening, 100%	Serosurvey, 50%; individual screening, 80%
Costs related to staff	See Supporting Tables S2 and S3	−50%–+100%
Costs related to transportation	US$0.11/km	+200%
Cost of the indirect IgG ELISA kit	US$470 per kit	±50%
Assumed useful lifespan of the capital equipment	See Supporting Table S1	±30%
Per diems given to teachers	None	US$60 per school
Number of ELISA plates a laboratory technician can run per day	2	1–3

We assumed that an additional visit to the selected schools would be necessary before the sampling to conduct community sensitization and to collect informed consent from the children’s parents; this visit would consist of a doctor visiting each school for 1 d (Figure 2).

#### School-based individual screening design

For this, we assumed that the school-based individual screening would be conducted at every government primary school within the province and would target 9-year-olds only (Table 1 and Figure 1). Based on data from the general statistics office of Vietnam,^21^ we estimated that there are 243 primary schools within an average province in Vietnam, each with around 88 children that are 9 y of age (Table 1). This results in a total sample size of 21 384 children per province.

For this strategy, we assumed that the community sensitization for collecting the blood samples would be incorporated into the community sensitization for administering the vaccine itself and therefore would not incur an additional cost (Figure 2).

### Sample collection and processing

#### Sample collection

We assumed that a sample collection team for one school would consist of six people (four nurses, one local doctor and one field supervisor) (Figure 2). We assumed that each sample collection team would require two vehicles and that they would sample one school per day, delivering the samples to a central laboratory within the province at the end of each day (Figure 2 and Table 1).

#### Sample processing and testing

We assumed that after delivery to the central laboratory, the samples would be stored overnight and processed the next morning. Based on interviews with local laboratory staff, we estimated that this pre-test processing would take approximately 6 h for the 81–88 samples collected from each school. The samples would then be stored in a freezer (−20°C) until they were tested.

Based on the WHO’s dengue serosurvey guide,^22^ we considered that the samples should be tested with an indirect immunoglobulin G (IgG) enzyme-linked immunosorbent assay (ELISA) and assumed it would be necessary to use a commercially available indirect ELISA kit, for which there is one currently available for detecting past dengue infections. The laboratory personnel and equipment required to perform the indirect ELISA were estimated through interviews with laboratory staff and by reviewing the indirect ELISA protocol; we assumed that in 1 workday one laboratory technician could run two ELISA plates (each plate containing up to 91 samples and the necessary controls). Subsequently, we estimated that 16 workdays would be required to process and test the samples for a school-based serosurvey of a typical province, while 326 workdays would be needed to process and test the samples if school-based individual screening was performed.

### Cost estimations

The financial costs were estimated using an ingredients-based costing approach, as recommended by the WHO’s costing guidelines.^25^ The cost analysis was performed from the health care provider’s perspective.

The costs of the laboratory supplies and equipment needed to perform the indirect ELISA, including that of the commercial kit, were determined from retail prices obtained from the suppliers used by the Oxford University Clinical Research Unit in Ho Chi Minh City. A wastage factor of 10% was applied to all relevant items. The assumed costs related to transportation (US$0.11/km) were based on the typical reimbursement rates for using a Vietnamese organization’s vehicle. The annual salary and bonuses/allowances rates for the different personnel were obtained from a hospital in Ho Chi Minh City and were converted to a daily staff cost, assuming 250 working days per year and an 8-h workday. All costs were adjusted to US dollars using the average 2016 exchange rate of 21 935 Vietnamese dong (VND) to US$1.^26^

The value of capital resources, here defined as laboratory equipment that lasts longer than 1 y, was annualized over its useful lifespan using straight-line depreciation (Supporting Table S1 and Box 1),^27^ that is, the replacement cost of the capital resource was divided by its useful lifespan, resulting in an annualized cost. For the serosurvey, we applied 5% of the annualized equipment cost to our cost estimates, as the majority of the province-level central laboratories should have the necessary laboratory equipment, which would be used for other activities throughout the year. For the more intensive individual screening strategy, we applied 100% of the annualized equipment cost to our cost estimates, based on the assumption that additional laboratory equipment would be necessary to process the required number of samples for this strategy. The same proportions were applied for equipment maintenance and calibration costs. Full details of the unit costs and the assumed quantity of inputs are presented in Supporting Tables S2 and S3.

### Outputs and sensitivity analyses

Our primary reported outcomes for both the serosurvey and individual screening strategies were the estimated total cost per province, the total cost per school and the cost per child sampled.

We performed a series of one-way sensitivity analyses to determine the robustness of the cost estimations and to assess to what extent the costs vary when a specific parameter or assumption changes. A summary of the sensitivity analyses is shown in Table 2.

## Results

### Costs of performing a school-based dengue serosurvey

**Figure 3.**
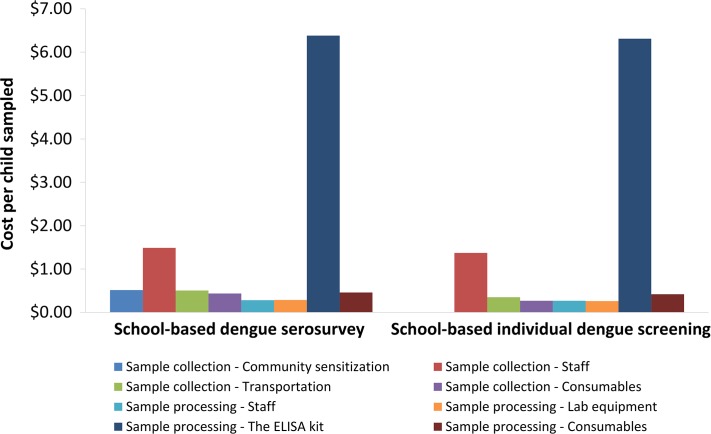
The projected costs stratified by input type. Costs are based on 2016 prices.

We estimated that the survey design described would cost US$10 074 for a typical province. This corresponds to US$840 per school sampled and US$10.36 per child sampled; 28% of the cost was related to sample collection with the remaining 72% related to sample processing and testing. A breakdown of the cost by input type is shown in Table 3. The key drivers were the cost of the ELISA kit and the costs relating to the personnel for sample collection (Figure 3).

**Table 3.** Projected cost of performing a school-based dengue serosurvey for a typical province

	Total cost per province (US$)	Total cost per school sampled (US$)	Total cost per child sampled (US$)
Sample collection			
Community sensitization	500.81	41.73	0.52
Staff	1447.80	120.65	1.49
Transportation	492.36	41.03	0.51
Consumables	425.72	35.48	0.44
Subtotal	2866.70	238.89	2.95
Sample processing and testing			
Staff	275.49	22.96	0.28
Lab equipment	280.23	23.35	0.29
ELISA kit	6204.00	517.0	6.38
Consumables	447.95	37.33	0.46
Subtotal	7207.67	600.64	7.42
Overall total	10 074.36	839.53	10.36

Costs are in 2016 prices.

### Costs of performing school-based individual dengue screening

The estimated cost of performing the individual dengue screening strategy was US$197 827 for a typical province per vaccination round. This corresponds to US$814 per school sampled and US$9.25 per child sampled. A breakdown of the cost by input type is shown in Table 4 and Figure 3.

**Table 4.** Projected cost of performing school-based individual dengue screening of 9-year-old children for a typical province

	Total cost per province (US$)	Total cost per school sampled (US$)	Total cost per child sampled (US$)
Sample collection			
Staff	29 318.02	120.65	1.37
Transportation	7477.78	30.77	0.35
Consumables	5778.44	23.78	0.27
Subtotal	42 574.23	175.20	1.99
Sample processing and testing			
Staff	5753.45	23.68	0.27
Lab equipment	5604.52	23.06	0.26
ELISA kit	134 937.00	555.30	6.31
Consumables	8958.02	36.86	0.42
Subtotal	155 252.99	638.90	7.26
Overall total	197 827.21	814.10	9.25

Costs are in 2016 prices.

### Sensitivity analyses

**Figure 4.**
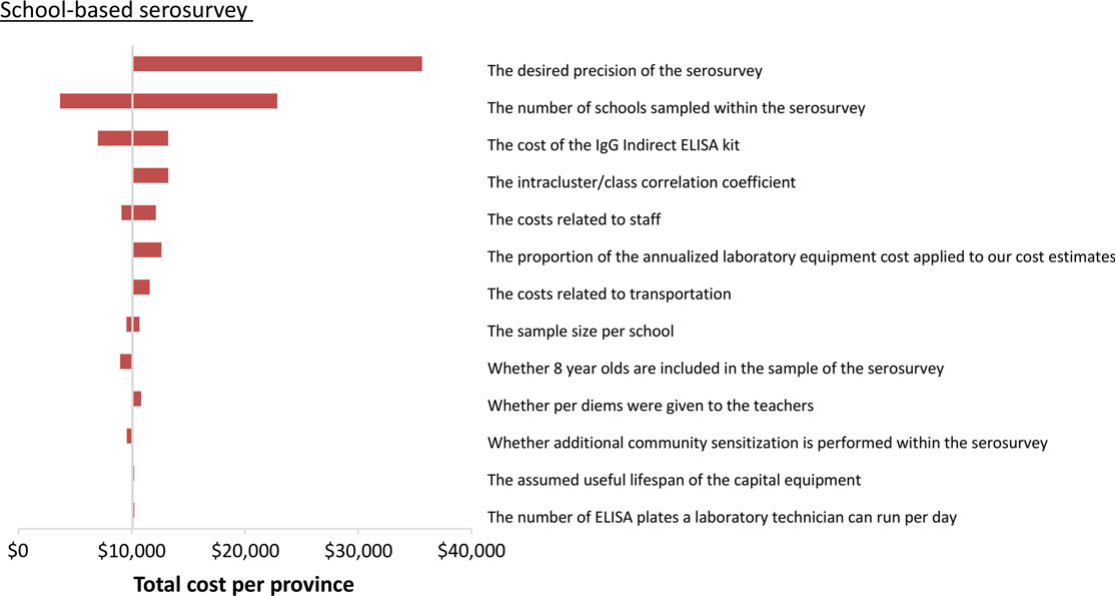
Tornado plot illustrating the impact of the sensitivity analysis on the estimated costs of performing a school-based serosurvey. The ranges investigated are shown in Table 2.

**Figure 5.**
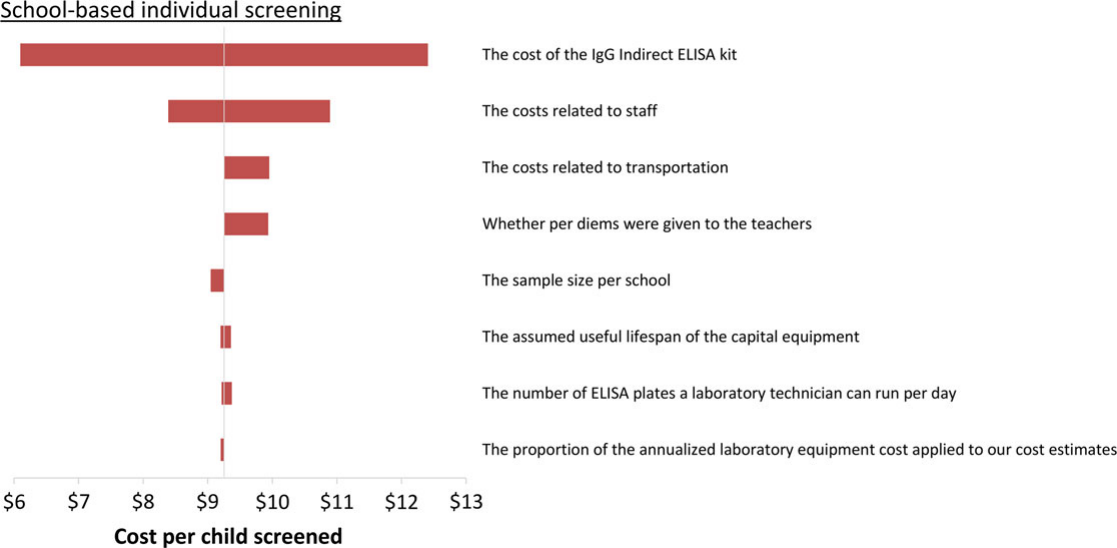
Tornado plot illustrating the impact of the sensitivity analysis on the estimated costs of performing school-based individual dengue screening of 9-year-old children. The ranges investigated are shown in Table 2.

The sensitivity of the estimated costs is illustrated in the tornado diagrams in Figures 4 and 5, which demonstrate the change in the costs when specific parameters/assumptions are altered. The projected cost of the serosurvey was most sensitive to changes in the number of schools sampled (which can be influenced by the parameters of the sample size calculation), the cost of the ELISA kit, staff costs and the proportion of the annualized equipment cost that was applied to the serosurvey (Figure 4). The projected cost of school-based individual screening was also sensitive to the assumed cost of the ELISA kit and staff costs (Figure 5).

The projected cost per child tested was inversely related to the number sampled/screened (Figure 5 and Supporting Figure S1). This was because some of the costs were either fixed or stepped-fixed (Box 1)^28^. As a result, the total cost of the serosurvey/individual screening was not linearly related to the total number sampled. For example, reducing the assumed number of children sampled per school within the serosurvey by 10% reduces its total cost by only 5% (Figure 4 and Supporting Figure S1).

## Discussion

As of April 2018, the SAGE is recommending that the preferred option for countries considering using Dengvaxia is a pre-vaccination screening strategy.^20^ It is therefore essential that the costs of such screenings are considered in future cost-effectiveness analyses informing how and where the vaccine should be used.

In this study we estimated that using a commercially available laboratory-based indirect IgG ELISA test to conduct large-scale school-based dengue screening of 9-year-olds would cost approximately US$197 827 for a typical province in Vietnam, or approximately US$9.25 per child sampled (Table 4). In fact, the potential need for screening when administering Dengvaxia was under debate even before the recent change in the WHO’s position.^11,14–16,29–32^ It should be noted that the SAGE did not initially consider large-scale dengue screening generally advisable, in part because of the complex logistical challenges of this approach as well as the potential financial constraints. Although individual screening is now being recommended, these logistical and financial challenges remain considerable.^16,20^

A potential alternative to performing the screening and vaccination within schools would be to use local health clinics. However, the costs of this approach and the best way to implement it on a large scale requires further investigation. Although screening might not be targeted at children only, achieving good coverage of the main population of interest would likely be more difficult than in the school environment. In addition, batch processing of samples in a central laboratory would still be necessary and the logistics of sample transport from multiple locations would likely inflate costs.

The development of rapid and reliable point-of-care screening tests to identify prior dengue infection could significantly reduce the costs of performing both school-based and clinic-based individual screening, as the testing could be performed immediately before vaccine administration, eliminating the need to transfer samples to a central laboratory (22% [US$1.99] of the estimated cost of performing screening). Point-of-care screening would also be more programmatically feasible on a large scale and should improve vaccine uptake. Repeated testing of initially seronegative individuals would also be possible. Finally, depending on the unit cost of point-of-care screening tests, this strategy might prove cheaper than using ELISA kits by eliminating sample transport and laboratory processing/personnel costs. Indeed, tests that are not point-of-care but are faster and less labour intensive than the indirect ELISA technique could still be advantageous. Unfortunately, the currently available rapid diagnostic tests for dengue typically focus on detecting acute infections and often have low sensitivity.^20,22,33^ This work further emphasizes the need to fund the further development of these tests.^7,34^

Serosurveys could still have an important role in identifying high-risk areas within a particular country. Given the high costs associated with individual screening, it may be more cost effective to conduct serosurveys first and then target the screening-based vaccination strategy to high-burden settings rather than targeting all areas, including low-burden settings where few individuals would be eligible for the vaccine. Serosurveys could also be useful for defining the age group(s) that should be targeted for screening, as this will vary depending on local transmission intensity. In addition, statistical models could be used to infer seroprevalence from age-stratified incidence data, reducing the need for serosurveys in multiple locations.^6^

In our study we estimated that a school-based dengue serosurvey for a typical province in Vietnam would cost US$10 074, assuming one classroom from the third, fourth and fifth grades at each of 12 schools were sampled, although ultimately the cost of performing such serosurveys depends on the chosen sample size and administration unit (Figure 4). The sample size and subsequent cost of the serosurvey were very sensitive to the level of desired precision (i.e., the width of the confidence interval around the estimated mean seroprevalence). If this is reduced from 10% to 5%, the number of schools that would need to be sampled would increase from 12 to 44 (Table 2), significantly increasing the projected cost of the serosurvey from US$10 074 to US$35 647 (Figure 4). Future studies are needed to explore the trade-offs between accuracy and the cost of these serosurveys.

It is important to restate that this costing is hypothetical and used Vietnam as an example. The costs of both serosurveys and individual screening will inevitably vary across different settings, depending on multiple factors such as the choice of the administration unit for the survey, if it is an urban or rural setting, which age groups are targeted, where the samples are processed and the method of community sensitization. In addition, staff costs will depend on the country, which staff are included in the sample collection team and whether there is an external project partner.

Our analysis has several limitations. We focused on quantifying financial costs and did not try to quantify the opportunity costs of donated resources, such as the use of Ministry of Health laboratory space or the economic value of the schoolteachers’ time (economic costs). It was also not possible to quantify the costs related to utilities. In addition, the staff salary/allowance rates we used were obtained from a hospital in Ho Chi Minh City and may be different in other provinces in Vietnam. We also assumed that the laboratory technicians would have sufficient training to perform the indirect ELISA technique; however, additional training might be required, incurring a cost not captured in this study. Finally, parents may refuse permission for their child to be tested, and further studies are needed to quantify this.

Another important issue to consider is the choice of the indirect ELISA technique to assess prior dengue exposure. Cross-reactivity with other flaviviruses and certain flavivirus vaccines is well recognized, potentially resulting in false-positive results, and WHO’s dengue serosurvey guide recommends that plaque reduction neutralization tests (PRNT) should be undertaken on a subgroup of samples to allow the indirect IgG ELISA assay cut-offs to be tailored to the local context.^22^ Although this would be important in Vietnam, where both Japanese encephalitis (JE) and Zika virus transmission are known to occur and JE vaccination is now widespread, we did not include this in our analysis, as it would be a one-off cost incurred for the whole country rather than for each province individually.

Even when using an individual screening strategy, there are important ethical considerations surrounding the use of Dengvaxia. Ultimately each endemic country will have to decide how and where to use this vaccine, in line with their own ethical evaluation.

We assumed that it would be necessary to use commercial ELISA kits in order to process the required number of samples, and this cost was found to be a key component of the total costs for both study designs (Figure 3). However, the cost of the ELISA kit may well change over time and could vary depending on demand, with potential discounts for larger orders. Furthermore, other settings may choose not to use commercial ELISA kits or alternative types of tests might be chosen.^22^ In this event additional studies would be needed to quantify the costs of alternative testing strategies, including costs for any validation that might be necessary.

While low- and lower middle–income countries may receive financial support from international agencies or philanthropic bodies to procure vaccines, funding is rarely made available to cover operational costs, such as for implementing serosurveys or performing individual screening. This highlights the importance of quantifying these costs and making efforts to reduce them.

It has been suggested that for seropositive recipients only one vaccine dose may be sufficient to induce immunity (as opposed to the currently recommended three-dose regime).^35^ If this is true, it could reduce the total cost of a screening-based vaccine administration strategy.^36^ However, the evidence that one vaccine dose is sufficient in seropositive recipients is preliminary and needs further investigation.^35^

## Conclusions

This study illustrates that deployment of Dengvaxia on a large scale will incur noteworthy operational costs associated with screening individuals and/or identifying high-burden areas. It is important that these operational costs are captured in future cost-effectiveness analyses informing how and where the vaccine is used. Globally, dengue causes significant health and economic burdens and in high seroprevalence settings, Dengvaxia could have a significant public health impact.

Our work further emphasizes the need to fund the further development of more rapid screening tests for past dengue infection.

The costs of dengue screening and serosurveys should be assessed in the context of the costs related to the unintended severe disease outcomes if the vaccine is used inappropriately. It is also important to consider the negative indirect effects these cases could have on the public’s perception of the safety of vaccines in general.

## Supplementary data

Supplementary data are available at Transactions online (http://trstmh.oxfordjournals.org/).

## Supplementary Material

Supplementary Data
