# Supplementary material for: Projected costs associated with school-based screening to inform deployment of Dengvaxia: Vietnam as a case study
Source: Trans R Soc Trop Med Hyg. 2018 Jul 3;112(8):369–77. doi: 10.1093/trstmh/try057 (PMC6092611; doi:10.1093/trstmh/try057)
Supplement: Supplementary Data [file try057_dengue_screening_paper_supporting_info.pdf]

Supporting information

Supporting Table S1: Assumed useful lifespan of the capital equipment

Equipment	Lifespan (years)
Centrifuge	5
Label printer	2
Plate washer	5
Plate reader	5
Autoclave	10
Single-channel pipette	2
Multi-channel pipette	2
Plate incubator	5
Freezer	5

Supporting Table S2: The assumed inputs and unit costs for the school-based serosurvey

Item	Total quantity	Unit cost (VND)	Unit cost (US$)
**Community sensitization**			
Local doctor (Workdays)	12	445,824	20.14
Travel (km)	2,520	2,500	0.11
Parental consent form	243	140	0.01
**Specimen collection**			
Staff			
Nurse (Workdays)	48	428,896	19.55
Local doctor (Workdays)	12	465,440	21.22
Field supervisor (Workdays)	12	465,440	21.22
Transportation			
Travel (km)	4,320	2,500	0.11
Consumables			
Lab coats	6	280,000	12.65
Tube holding rack	6	70,000	3.16
Alcohol container	6	30,000	1.36
Waste collection box	3	165,000	7.45
Tourniquet	9	8,000	0.36
Marker pen/pen	6	25,000	1.13
Cool pack	36	5,000	0.23
Sample transport box	6	120,000	5.42
Ethanol (ml)	1,069	22	0.001
Waste collection bag	108	1,300	0.06
Sharp container	4	30,000	1.36
Hand sanitizer (ml)	360	176	0.01
Cotton ball	2,138	16	0.001
Needle	972	810	0.04
Vacutainer holder	10	250	0.01
Blood collection tube	1,069	1,470	0.07
Bandaid	1,069	480	0.02
Collection tube label	1,069	20	0.001
Specimen collection form	49	140	0.01
Latex gloves (pair)	288	1,554	0.07
Disposable syringe	1,069	810	0.04
Register book	0.4	24,000	1.08
Absorbent paper (roll)	12	44,000	1.99
Bleach solution (ml)	72	6,600	0.30
**Specimen processing and testing**			
Staff			
Specimen reception/processing - Lab technician (Work days)	9	387,360	17.66
Specimen testing - Lab technician (Work days)	7	387,360	17.66
Equipment[a]			
Serum separation centrifuge	1	1,194,000	54.43
Label printer	1	112,500	5.13
Plate washer	1	935,000	42.63
Plate reader	1	1,737,400	79.21
Autoclave	1	597,842	27.26
Single-channel pipettes (200 µl, 1000 µl)	2	119,500	5.45
Multi-channel pipette	1	358,750	16.36
Plate incubator	1	459,261	20.94
Freezer for specimen storage	1	113,000	5.15

Instrument maintenance cost	1	400,000	18.24
Consumables			
Specimen reception/processing - Hand sanitizer/ (ml)	48	176	0.01
Specimen reception/processing - Gloves	24	15,540	0.70
Specimen reception/processing - Register book	0.1	24,000	1.08
Specimen reception/processing - Specimen shipment form	49	140	0.01
Specimen reception/processing - Latex gloves (pair)	48	15,540	0.70
Specimen reception/processing - Pipette (disposable)	1,069	1,062	0.05
Specimen reception/processing - Cryotube	1,069	3,520	0.16
Specimen reception/processing - Cryotube label	1,069	15	0.0007
Specimen reception/processing - 81 tube box	12	100,000	4.52
Specimen reception/processing - Autoclave bag	24	1,300	0.06
Specimen reception/processing - Waste bin	2	165,000	7.45
Specimen reception/processing - Register book	0.4	24,000	1.08
Specimen reception/processing - Marker	12	2,500	0.11
Specimen testing - Hand sanitizer (ml)	48	176	0.01
Specimen testing - Latex gloves (pair)	48	1,554	0.07
Specimen testing - Lab coat for technician	1	280,000	12.65
Specimen testing - Discard beaker	2	35,000	1.58
Specimen testing - Bleach solution (ml)	24	6,600	0.30
Specimen testing - Absorbent paper	2	44,000	1.99
Specimen testing - Dilution tray	48	21,895	0.99
Specimen testing - Pipette tips (200 µl)	1,604	168	0.01
Specimen testing - Pipette tips (1000 µl)	120	189	0.01
Specimen testing - Distilled water (ml)	24,000	3	0.0001
Specimen testing - ELISA kit	13	10,405,236	470.00
Specimen testing - Register book	0.1	24,000	1.08
Specimen testing - Waste bag	36	1,300	0.06
Sample decontamination - Waste disposal	1	40,000	1.81

VND: Vietnamese dong. [a] The unit costs for the laboratory equipment have been annualized and account for the proportion the equipment would be used for used for the serosurvey (5%). Values are in 2016 prices.

Supporting Table S3: The assumed inputs and unit costs for the school-based individual screening strategy

Item	Total quantity	Unit cost (VND)	Unit cost (US$)
**Specimen collection**			
Staff			
Nurse (Workdays)	972	428,896	19.55
Local doctor (Workdays)	243	465,440	21.22
Field supervisor (Workdays)	243	465,440	21.22
Transportation			
Travel (km)	65,610	2,500	0.11
Consumables			
Lab coats	12	280,000	12.65
Tube holding rack	12	70,000	3.16
Alcohol container	12	30,000	1.36
Waste collection box	6	165,000	7.45
Tourniquet	18	8,000	0.36
Marker pen/pen	12	25,000	1.13
Cool pack	36	5,000	0.23
Sample transport box	12	120,000	5.42
Ethanol (ml)	23,522	22	0.001
Waste collection bag	2,187	1,300	0.06
Sharp container	73	30,000	1.36
Hand sanitizer (ml)	7,290	176	0.01
Cotton ball	47,045	16	0.001
Needle	21,384	810	0.04
Vacutainer holder	214	250	0.01
Blood collection tube	23,522	1,470	0.07
Bandaid	23,522	480	0.02
Collection tube label	23,522	20	0.001
Specimen collection form	1,069	140	0.01
Latex gloves (pair)	5,832	1,554	0.07
Disposable syringe	23,522	810	0.04
Register book	7	24,000	1.08
Absorbent paper (roll)	243	44,000	1.99
Bleach solution (ml)	1,458	6,600	0.30
**Specimen processing and testing**			
Staff			
Specimen reception/processing - Lab technician (Work days)	182	387,360	17.66
Specimen testing - Lab technician (Work days)	144	387,360	17.66
Equipment[a]			
Serum separation centrifuge	1	23,880,000	1088.67
Label printer	1	2,250,000	102.58
Plate washer	1	18,700,000	852.52
Plate reader	1	34,748,000	1584.13
Autoclave	1	11,956,833	545.10

Single-channel pipettes (200 µl, 1000 µl)	1	2,390,000	108.96
Multi-channel pipette	2	7,175,000	327.10
Plate incubator	1	9,185,220	418.75
Freezer for specimen storage	1	2,260,000	103.03
Instrument maintenance cost	1	8,000,000	364.71
<u>*Consumables*</u>			
Specimen reception/processing - Hand sanitizer/ (ml)	972	176	0.01
Specimen reception/processing - Gloves	486	15,540	0.70
Specimen reception/processing - Register book	2	24,000	1.08
Specimen reception/processing - Specimen shipment form	1,069	140	0.01
Specimen reception/processing - Latex gloves (pair)	972	15,540	0.70
Specimen reception/processing - Pipette (disposable)	23,522	1,062	0.05
Specimen reception/processing - Cryotube	23,522	3,520	0.16
Specimen reception/processing - Cryotube label	23,522	15	0.001
Specimen reception/processing - 81 tube box	264	100,000	4.52
Specimen reception/processing - Autoclave bag	486	1,300	0.06
Specimen reception/processing - Waste bin	2	165,000	7.45
Specimen reception/processing - Register book	7	24,000	1.08
Specimen reception/processing - Marker	12	2,500	0.11
Specimen testing - Hand sanitizer (ml)	972	176	0.01
Specimen testing - Latex gloves (pair)	972	1,554	0.07
Specimen testing - Lab coat for technician	1	280,000	12.65
Specimen testing - Discard beaker	2	35,000	1.58
Specimen testing - Bleach solution (ml)	486	6,600	0.30
Specimen testing - Absorbent paper	49	44,000	1.99
Specimen testing - Dilution tray	972	21,895	0.99
Specimen testing - Pipette tips (200 µl)	35,284	168	0.01
Specimen testing - Pipette tips (1000 µl)	2,430	189	0.01
Specimen testing - Distilled water (ml)	486,000	3	0.0001
Specimen testing - ELISA kit	287	10,405,236	470.00
Specimen testing - Register book	2	24,000	1.08
Specimen testing - Waste bag	729	1,300	0.06
Sample decontamination - Waste disposal	11	40,000	1.81

VND: Vietnamese dong. [a]The unit costs for the laboratory equipment have been annualized and account for the proportion equipment would be used for used for the screening (100%). Values are in 2016 prices.

School-based serosurvey

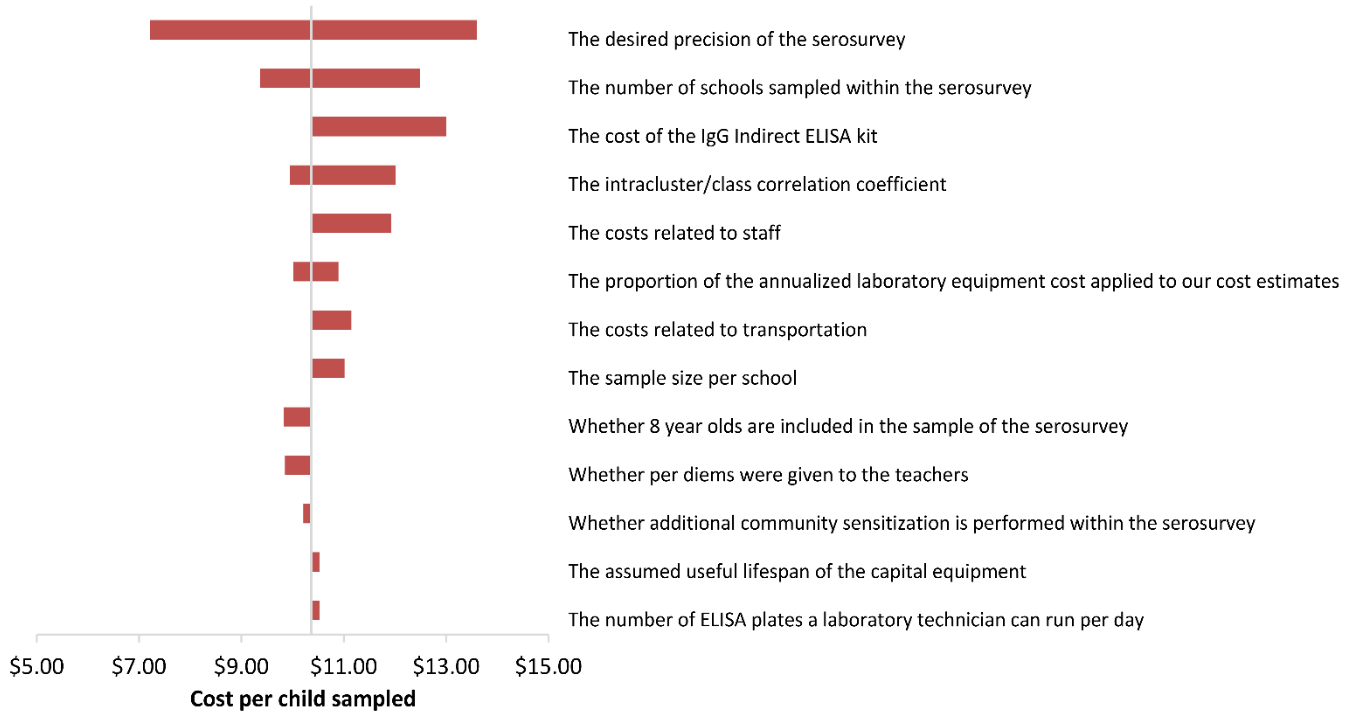

Supporting Figure S1: Tornado plot illustrating the impact of the sensitivity analysis on the estimated costs per test of the school-based serosurvey. *The ranges investigated are shown in Table 2.*
